# Hybrid surgery versus endovascular intervention for patients with chronic internal carotid artery occlusion: A single-center retrospective study

**DOI:** 10.3389/fsurg.2022.976318

**Published:** 2022-09-02

**Authors:** Tao Sun, Yiming He, Fei Wang, Bo Mao, Mengtao Han, Peng Zhao, Wei Wu, Yunyan Wang, Xingang Li, Donghai Wang

**Affiliations:** ^1^ Department of Neurosurgery, Qilu Hospital of Shandong University, Cheeloo College of Medicine and Institute of Brain and Brain-Inspired Science, Shandong University, Jinan, China; ^2^ Jinan Microecological Biomedicine Shandong Laboratory and Shandong Key Laboratory of Brain Function Remodeling, Jinan, China; ^3^ Department of Neurology, Qilu Hospital of Shandong University, Cheeloo College of Medicine, Shandong University, Jinan, China; ^4^ Department of Neurosurgery, Qilu Hospital of Shandong University Dezhou Hospital (Dezhou, China), Cheeloo Hospital of Shandong University, Jinan, China

**Keywords:** carotid artery occlusion, hybrid surgery, endovascular intervention, recanalization, carotid endarterectomy (CEA)

## Abstract

**Objective:**

Chronic internal carotid artery occlusion (CICAO) can cause transient ischemic attack (TIA) and ischemic stroke. Carotid artery stenting (CAS) with embolic protection devices and hybrid surgery combining carotid endarterectomy and endovascular treatment are effective methods for carotid revascularization. The objective of this study was to evaluate and compare the effect and safety of the two surgical procedures.

**Methods:**

This was a single-center retrospective study. In this study, 44 patients who underwent hybrid surgery and 35 who underwent endovascular intervention (EI) at our center were enrolled consecutively between May 2016 and March 2022. All patients were classified into four groups (A-D), as described by Hasan et al. We recorded and analyzed clinical data, angiographic characteristics, technical success rate, perioperative complications, and follow-up data.

**Results:**

There was no significant difference in baseline characteristics between hybrid surgery group and EI group, except for plasma high density lipoproteins (HDL) levels (median [interquartile range]: hybrid surgery, 0.99 [0.88–1.18] vs. EI, 0.85 [0.78–0.98] mmol/L, *P* = 0.001). The technical success rate of hybrid surgery was higher than that of EI (37/44 [84.1%] vs. 18/35 [51.4%], *P* = 0.002; type A: 15/16 [93.8%] vs. 10/11 [90.9%], *P* = 1.000; type B: 9/10 [90.0%] vs. 5/7 [71.4%], *P* = 0.537; type C: 12/15 [80.0%] vs. 3/12 [25.0%], *P* = 0.004; type D: 1/3 [33.3%] vs. 0/5 [0%], *P* = 0.375). No significant difference was observed in the incidence of perioperative complications between the two procedures (hybrid surgery: 7/44 [15.9%] vs. EI: 6/35 [17.1%], *P* = 0.883). In addition, there were no significant differences in the rates of stroke and restenosis during follow-up.

**Conclusions:**

For patients with symptomatic CICAO, hybrid surgery may have an advantage over EI in successfully recanalizing occluded segments. There was no significant difference in safety and restenosis between hybrid surgery and EI.

## Introduction

Internal carotid artery occlusion (ICAO) is a major cause of ischemic stroke. Chronic ICAO (CICAO) is usually defined as total occlusion of the ICA for at least four weeks on an angiogram (1). Symptomatic patients with CICAO have a high annual recurrent stroke rate of 6%–20% (2, 3). Currently, the best medical therapy (BMT), including the combination of lipid-lowering, anti-platelet, and blood pressure-modifying agents, remains the mainstay of treatment for CICAO (1). Surgical recanalization can be indicated in patients with recurrent ischemic symptoms during regular drug therapy. A multicenter, prospective, randomized controlled study indicated that symptomatic patients with CICAO did not benefit from extracranial-intracranial (EC-IC) artery bypass (4) Thus, carotid endarterectomy (CEA) and endovascular interventions (EI) are potential but challenging choices. Due to technical difficulty and unfavorable outcomes in early clinical trials, society guidelines failed to recommend carotid revascularization surgery for patients with CICAO (5, 6).

In recent years, some studies have reported considerable progress in carotid recanalization with advancements in technology. On the one hand, EI with distal and proximal protective devices has been proven to be feasible and safe in recanalizing the occluded ICA (5, 7, 8). On the other hand, hybrid surgery seems to be a more promising and advantageous approach in the treatment of CICAO (9, 10). However, the small sample size and lack of control groups limit the broad applicability of the hybrid technique in CICAO. We reviewed the medical records of patients with CICAO who underwent carotid artery recanalization at our center. A comparative study between EI and hybrid recanalization was performed to investigate the technical success rate, security, clinical characteristics, complications, and outcomes.

## Materials and methods

We conducted a single-center, observational, retrospective cohort study of patients with symptomatic CICAO who underwent EI or hybrid recanalization at the Qilu Hospital of Shandong University. This study was approved by the ethics committee of our hospital. We accessed electronic medical records to evaluate eligibility and collected perioperative data from eligible subjects.

### Patients and materials

**Figure 1:**
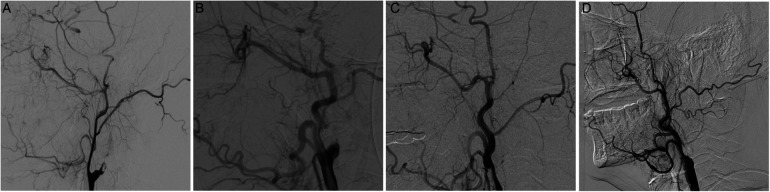
Illustration of the Hasan’ classification of chronic internal carotid artery occlusion (CICAO). (**A**) Type A, taper stump; cavernous and/or petrous segments with collateral filling. (**B**) Type B, non-taper stump; cavernous and/or petrous segments with collateral filling. (**C**) missing stump; cavernous and/or petrous segments with collateral filling. (**D**) Type D: cavernous and/or petrous segments without collateral filling.

A total of 79 symptomatic patients with CICAO who underwent EI or hybrid recanalization between May 2016 and March 2022 were enrolled. The exact inclusion criteria were as follows: Complete occlusion of the internal carotid artery (ICA) lasting for at least 4 weeks after diagnosis by computed tomography angiography (CTA), magnetic resonance angiography (MRA), or digital subtraction angiography (DSA); recurrent ipsilateral ischemic symptoms (amaurosis fugax, transient ischemic attack [TIA], or ischemic stroke) despite medical treatment (dual antiplatelet); no ipsilateral intracranial artery stenosis except the intracranial segment of the ICA; no intracranial smoke-like blood vessel. TIA is defined as a transient focal neurological symptom without acute infarction. The definition of minor stroke is a new, nondisabling neurological deficit with a ≤3-point increase in the National Institutes of Health Stroke Scale (NIHSS) score. An increase in NIHSS score of more than 3 points were classified as major stroke (11, 12). Patients with severe system disease who could not tolerate surgery and anesthesia were excluded. All patients underwent DSA to confirm complete occlusion of the ICA, collateral circulation, occlusion stump shape, and location of blood reflux (level of distal ICA reconstitution). The patients were divided into four groups according to the criteria described by Hasan et al. (13). Figure 1 depicts the details of Hasan's classification. Computed tomography perfusion (CTP) imaging was performed in all patients to evaluate cerebral perfusion. All patients were deemed to have a standard risk of complications for both procedures. Additionally, both procedures were performed by interventionists and surgeons with adequate skill and experience. Data on venous blood samples, clinical and demographic characteristics, angiography findings, and outcomes were collected and reviewed independently by neurologists and interventionists.

### Hybrid surgery procedure

All hybrid surgical procedures were performed in a hybrid operating room. Dual antiplatelet therapy and general anesthesia were administered to all patients. First, an incision was made along the anterior border of the sternocleidomastoid muscle, and standard CEA was performed. After removing the plaque, a 4F Fogarty embolectomy balloon catheter was inserted into the distal true lumen to pull out the distal thrombus. Arterial sheath was inserted through a right femoral puncture using the Seldinger technique or a carotid incision. Subsequently an immediate intraoperative angiography was performed. Balloon dilation and stent implantation were applied as appropriate to resolve the stenosis, occlusion, and dissection. Technical success was defined as final residual diameter stenosis <20% and TICI grade 3 antegrade flow after recanalization of the occlusion.

### EI procedures

All EI procedures were performed using an 8-F femoral sheath under local anesthesia. Proximal or distal balloon protection devices were used to prevent distal embolism in all cases. Aspirin (100 mg) and clopidogrel (75 mg) were administered daily for at least 7 days before the procedure. A micro-guidewire and microcatheters were carefully used to cross the occluded segment. Once the wire entered the distal true lumen, the distal or proximal protective devices were deployed. Balloons and stents were used to reconstruct carotid arteries. Balloons can be used again to improve stents with inadequate expansion. The definition of technical success was consistent with that of hybrid surgery.

### Complications and follow-up

Perioperative complications, including mortality, intracerebral hemorrhage, ischemic stroke, cerebral hyperperfusion syndrome (CHS), and wound infection, were observed and recorded. CHS was defined as a severe ipsilateral headache, seizures, or intracranial hemorrhage (14). Patients were followed-up with CTA, MRA, or DSA. Restenosis is defined as a reduction in the diameter of the target artery by at least 70% (15, 16). Complications during follow-up, including mortality, stroke, and restenosis, were recorded.

### Statistical analysis

Statistical analysis was performed using SPSS software 25.0 (IBM Corp., New York, United States). Normally distributed continuous variables were expressed as mean ± standard deviation and were analyzed using Student's t-test. Abnormally distributed continuous variables are expressed as median (interquartile range [IQR]) and analyzed using the Mann-Whitney U test. Categorical variables were described as percentages and analyzed using the chi-square test or Fisher's exact test. A *P* value of <0.05 was considered statistically significant.

## Results

Among 79 participants who were enrolled in the study, 44 underwent hybrid surgery and 35 underwent EI. The baseline characteristics of the participants in the two groups are summarized in Table 1. Compared with patients undergoing EI, patients undergoing hybrid surgery had higher serum levels of High-density lipoproteins cholesterol (HDL-C) (median [interquartile range]: 0.99 [0.88–1.18] vs. 0.85 [0.78–0.98] mmol/L, *P* = 0.001, Table 1). However, no significant differences in age, sex, symptom, hypertension, diabetes mellitus, coronary heart disease, smoking history, alcohol drinking, serum total cholesterol, low-density lipoprotein cholesterol (LDL-C), triglyceride, homocysteine (hCY), glucose, uric acid, and creatinine were found between the two groups (*P* ≥ 0.05, Table 1).

**Table 1:** Baseline characteristics of study participants.

Demographic, clinical, and laboratory items	All cases	Hybrid surgery group	EI group	*P-*value (<0.05)
No. patients, *n*	79	44	35	–
Age, y	63.34 ± 6.88	63.59 ± 5.80	63.03 ± 8.11	0.731
Sex, male	71 (89.8%)	38 (86.4%)	33 (94.3%)	0.290
Symptom				
TIA, *n*	24 (30.4%)	13 (29.5%)	11 (31.4%)	0.857
Minor stroke, *n*	43 (54.4%)	26 (59.1%)	17 (48.6%)	0.351
Major stroke, *n*	12 (15.2%)	5 (11.4%)	7 (20.0%)	0.288
Total cholesterol, mmol/L	3.42 ± 0.89	3.57 ± 0.95	3.23 ± 0.79	0.096
HDL-C, mmol/L	0.91 (0.82–1.12)	0.99 (0.88–1.18)	0.85 (0.78–0.98)	0.001
LDL-C, mmol/L	1.83 (1.48–2.25)	2.00 (1.55–2.39)	1.69 (1.35–2.24)	0.111
Triglyceride, mmol/L	1.24 (1.02–1.61)	1.23 (1.03–1.60)	1.28 (0.97–1.69)	0.664
hCY, μmol/L	13.40 (11.70–17.30)	13.25 (11.00–17.08)	15.00 (11.90–17.50)	0.385
Serum glucose, mmol/L	5.58 (4.86–6.56)	5.54 (4.89–6.38)	5.60 (4.53–7.06)	0.941
Serum uric acid, μmol/L	286.00 (247.00–343.00)	280 (246.25–331.25)	289.00 (248.00–348.00)	0.374
Creatinine, μmol/L	72.03 ± 14.54	71.27 ± 13.48	72.97 ± 15.92	0.609
Diabetes mellitus, *n*	34 (43.0%)	19 (43.2%)	15 (42.9%)	0.977
Hypertension, *n*	51 (64.6%)	31 (70.5%)	20 (57.1%)	0.219
Coronary heart disease, *n*	14 (17.7%)	6 (13.6%)	8 (22.9%)	0.286
History of smoking, *n*	55 (69.6%)	29 (65.9%)	26 (74.3%)	0.421
History of drinking, *n*	38 (48.1%)	18 (40.9%)	20 (57.1%)	0.151

Data presented as mean ± standard deviation or median (IQR) based on normality of continuous variables. Data presented as *n* (%) for categorical variables. IQR, interquartile range; HDL-C, high-density lipoproteins cholesterol; LDL-C, low density lipoprotein cholesterol; hCY, homocysteine.

Lesion characteristics and technical success rates are shown in Table 2. The recanalization success rate was 69.6% at our center. The hybrid surgery group had a higher recanalization success rate than the EI group (37/44 [84.1%] vs. 18/35 [51.4%], *P* = 0.002, Table 2). There was no difference between the two patient groups in terms of lesion location and Hasan classification (*P* ≥ 0.05, Table 2). For types A and B, both hybrid surgery and EI had high recanalization success rates, and there was no difference between the two procedures (type A: 15/16 [93.8%] vs. 10/11 [90.9%], *P* = 1.000; type B: 9/10 [90.0%] vs. 5/7 [71.4%], *P* = 0.537; Table 2). For type C, the recanalization success rate was significantly higher in the hybrid surgery group (12/15 [80.0%] vs. 3/12 [25.0%], *P* = 0.004). Additionally, for type D, recanalization was difficult in both procedures (1/3 [33.3%] vs. 0/5 [0%], *P* = 0.375).

**Table 2:** Lesion characteristics and technical success rate.

	All cases	Hybrid surgery group	EI group	*P-*value (<0.05)
Lesion location, right/left, *n*		25/19	21/14	0.776
Hasan classification				
A, *n*	27 (34.1%)	16 (36.4%)	11 (31.4%)	0.646
Success rate in A, *n*	25 (92.6%)	15 (93.8%)	10 (90.9%)	1.000
B, *n*	17 (21.5%)	10 (22.7%)	7 (20.0%)	0.770
Success rate in B, *n*	14 (82.4%)	9 (90.0%)	5 (71.4%)	0.537
C, *n*	27 (34.2%)	15 (34.1%)	12 (24.3%)	0.986
Success rate in C, *n*	15 (55.5%)	12 (80.0%)	3 (25.0%)	0.004
D, *n*	8 (10.1%)	3 (6.8%)	5 (14.3%)	0.455
Success rate in D, *n*	1 (12.5%)	1 (33.3%)	0(0%)	0.375
Overall success rate	55 (69.6%)	37 (84.1%)	18 (51.4%)	0.002

Data presented as n (%) for dichotomous or categorical variables.

The incidence of perioperative complications, complications during follow-up and NIHSS score are shown in Table 3. The perioperative complication rates of hybrid surgery and EI were 15.9% and 17.1%, respectively (7/44 vs. 6/35, *P* = 0.883). In the hybrid group, one patient had hemiparesis after unsuccessful recanalization and returned to the baseline state with conservative treatment. Two patients had CHS. Cardiovascular events occurred in three patients, but only with elevated myocardial enzymes and returned to normal levels. One patient developed an incision infection, possibly due to long-term uncontrolled diabetes. In the EI group, CHS occurred in four patients and resulted in intracranial hemorrhage and death in one patient. Two patients had elevated levels of myocardial enzymes. No significant differences were found in perioperative complications and complications during follow-up. All patients were followed-up; the median (interquartile range) follow-up period was 24 (10–49) months. Patients who underwent hybrid surgery had longer follow-up, because of the earlier initiation of hybrid surgery than EI in our center. In the hybrid group, one patient died of a cardiovascular event. Recurrent stroke occurred in one unsuccessful patient; In addition, one patient had restenosis and one patient had re-occlusion. In the EI group, three patients with unsuccessful revascularization experienced a recurrent TIA or stroke, one of whom died. Three patients had restenosis and one patient had re-occlusion. The restenosis/re-occlusion rates in hybrid surgery and EI were 5.4% and 22.2%, respectively (2/37 vs. 4/18, *P* = 0.082). There were no significant differences between hybrid group and EI group in pre-operative and follow-up (exclusion of deaths) NIHSS score. Meanwhile, the NIHSS score did not improve significantly after procedure. This may be because that CEA and carotid artery stenting (CAS) are both aimed at preventing stroke, but it is difficult to improve the neurological deficits that have occurred.

**Table 3:** Incidence of perioperative complications, complications during follow-up and NIHSS score.

	All cases	Hybrid surgery group	EI group	*P-*value
Perioperative Complication				
Mortality	1 (1.3%)	0	1 (2.9%)	0.443
Stroke	1 (1.3%)	1 (2.3%)	0	1.000
Intracerebral hemorrhage	1 (1.3%)	0	1 (2.9%)	0.443
CHS	6 (7.6%)	2 (4.5%)	4 (11.4%)	0.398
Cardiovascular events	5 (6.3%)	3 (6.8%)	2 (5.7%)	1.000
Wound infection	1 (1.3%)	1 (2.3%)	0	1.000
Follow-up (months)	24 (10–49)	38 (12.25–53)	18 (9–30)	0.013
Complications during follow-up				
Mortality	2 (2.5%)	1 (2.3%)	1 (2.9%)	1.000
Recurrent TIA/stroke	4 (5.1%)	1 (2.3%)	3 (8.6%)	0.317
Restenosis in successful cases	4 (7.2%)	1 (2.7%)	3 (16.7%)	0.097
Re-occlusion in successful cases	2 (3.6%)	1 (2.7%)	1 (5.6%)	1.000
NIHSS score				
Pre-procedure	2.0 (0–3.0)	2 (0–2.0)	2 (0–3.0)	0.459
Follow-up	2.0 (0–3.0)	1 (0–2.0)	2 (0–3.0)	0.350

Data presented as mean ± standard deviation or median (IQR) based on normality of continuous variables. Data presented as *n* (%) for dichotomous or categorical variables. IQR, interquartile range; CHS, cerebral hyperperfusion syndrome; NIHSS, National Institutes of Health Stroke Scale.

### Example patient 1

**Figure 2:**
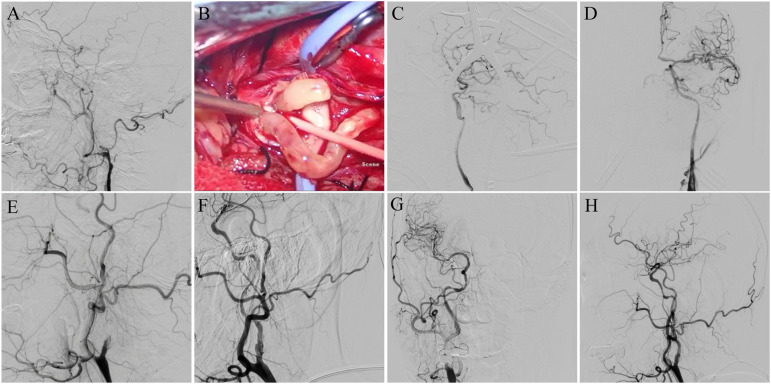
Example patients. (**A**) Preoperative digital subtraction angiogram (DSA) reveals a left type C CICAO. (**B**) A 4F Fogarty embolectomy balloon catheter is used to pull out the distal thrombus. (**C**) Microwire is crossed over the lesion, and carotid reconstruction is performed with balloons and stents. (**D**) DSA revealed successful revascularization. (**E**) Preoperative DSA reveals a right type A CICAO. (**F**) The carotid reconstruction is performed from distal to proximal with balloons and stents. (**G,H**) DSA reveals successful revascularization.

A 67-year-old woman complaining of glossolalia and right limb weakness for six months underwent hybrid surgery. Preoperative DSA revealed left type C CICAO (Figure 2A). A 4F Fogarty embolectomy balloon catheter was inserted into the distal lumen to pull out the distal thrombus (Figure 2B) after CEA, and a 6F arterial sheath was inserted through a partial suture incision. In addition, a Synchro 0.014-in. microwire (Stryker Corp., Michigan, United States) with an Echelon-10 microcatheter (Echelon Corp., California, United States) crossed over the lesion. Carotid reconstruction was performed using a Gateway 3.25 × 15 mm balloon (Boston Scientific Corp., Massachusetts, United States), Enterprise 4 × 39 mm (Johnson & Johnson Corp., New Jersey, United States), and Wallstent 7 × 40 mm (Boston Scientific Corp., Massachusetts, United States) stents. DSA revealed successful ICA revascularization (Figure 2D).

### Example patient 2

A 56-year-old man complaining of glossolalia for two months underwent EI. Preoperative DSA revealed right type A CICAO (Figure 2E). A Synchro 0.014-in. microwire (Stryker Corp., Michigan, United States) was advanced through an Echelon-10 microcatheter (Echelon Corp., California, United States) to penetrate the occluded segment. After the pre-dilatation of the stenosis with a 2 × 20 mm balloon, a Proender 5 mm distal embolic protection device (TjwyMedical Corp., Beijing, China) was deployed. A Viatrac 4 × 30 mm balloon (Abbott Corp., Chicago, United States) was used to dilate the stenosis gradually. Apollo 3 × 18 mm (MicroPort Corp., Shanghai, China), Apollo 3 × 13 mm, Apollo 3.5 × 13 mm, and Excel 14 × 28 mm stents (Bluesail Corp., Shandong, China) were deployed to reconstruct the carotid artery (Figures 2G,H).

## Discussion

It is estimated that 15,000 to 20,000 ischemic events due to ICAO occur annually in the United States (17). However, the natural history and clinical manifestations of ICAO vary. Most patients with ICAO are asymptomatic, probably because of less forward flow and establishment of collateral circulation (13). The remaining symptomatic patients with ICAO are biased toward non-benign outcomes such as insufficient cerebral perfusion, embolus detachment, and cognitive dysfunction (18). Therefore, patients who require hospitalization for surgical treatment are usually symptomatic. Due to the immaturity of early technology, CEA and endovascular treatment have not achieved satisfactory results such as a low recanalization success rate and high perioperative risk. However, with the application of embolic protection devices and the emergence of hybrid surgery, treatment outcomes improved in patients with ICAO (8, 9).

Several clinical trials comparing CAS and CEA, such as the Asymptomatic Carotid Trial (ACT I) and the Carotid Revascularization Endarterectomy versus Stenting Trial (CREST), have not only shown the effectiveness of revascularization for the treatment of patients with carotid stenosis but also showed no significant difference in the rate of the primary composite endpoint (stroke, myocardial infarction, or death) between the two procedures (19, 20). However, none of these trials included ICAO patients. Recently, the number of studies and reviews on ICAO has gradually increased. Both hybrid surgery and EI can recanalize an occluded carotid artery with a low rate of neurological complications. The technical success rate of EI was 55%–70%, while that of hybrid surgery was significantly increased to 66.0%–98% (1, 5). Despite these data, the assessment and treatment of patients with ICAO remain controversial.

EI has the advantages of being minimally invasive and without general anesthesia, but fibrotic thrombus and collapsed distal blood vessels make it difficult for the micro-guidewire to pass through the occlusive segment. Attempting violence and the use of a microwire with a stiffer tip and a catheter with a tapered tip will increase the risk of dissection and perforation (21, 22). Meanwhile, protecting the cerebral circulation from distal embolism during the procedure is indispensable. Several previous studies have demonstrated that types A and B are more suitable for EI because of visualization of the initial segment of the ICA at the common carotid artery bifurcation (23, 24). In our study, the technical success rates of types A and B were 90.9% and 71.4%, respectively. However, endovascular therapy encounters difficulties when treating type C lesions. The recanalization success rate was significantly reduced to 25.0% (Table 2). Vascular perforations, vessel ruptures, and new neurological deficits during the procedures were not observed in our series, perhaps because of the very gentle operation and the use of embolic protection devices.

CEA is the “gold standard” for carotid atherosclerosis treatment. However, CEA does not guarantee successful recanalization of the occluded carotid artery because of its inability to observe and treat distal tandem lesions. Thus, hybrid surgery, which can treat both proximal and distal lesions, has inherent advantages in the treatment of CICAO (22). CEA can remove plaque and build an artificial stump, which allows easier access of the guidewire to the distal artery and EI. Moreover, we attempted to insert the arterial sheath through a carotid incision in some patients, which allowed us to control the guidewire more easily. Our study also confirmed a higher technical success rate of hybrid surgery compared to EI, especially in type C patients. In addition, two types of thrombi were found during thrombus retraction, one of which was a soft dark red thrombus, while the other one was a tough fibrotic thrombus. We hypothesized that fibrotic thrombus reflected a longer occlusion time and that dissection was more likely to occur after thrombectomy.

In addition to technical success, the safety of CICAO treatment should be considered. In this study, there was no difference in the perioperative complications between the hybrid surgery and EI groups. Similar to previous reports (1), the perioperative complication rates of hybrid surgery and EI were 15.9% and 17.1%, respectively. The main perioperative complication in both groups was cerebral CHS, which resulted in intracranial hemorrhage and death in only 1 patient undergoing endovascular therapy. Therefore, strict postoperative management of blood pressure is necessary. Incision hematoma did not occur, but one patient developed an incision infection, probably due to long-term diabetes. The restenosis/re-occlusion rate of hybrid surgery was lower than that of endovascular treatment, but there was no statistical difference (2/37 [5.4%] vs. 4/18 [22.2%], *P* = 0.082). One patient who underwent hybrid surgery developed re-occlusion six months postoperatively, and subsequent endovascular therapy failed to recanalize the carotid artery. The cause of restenosis or re-occlusion in these patients needs to be further explored.

We acknowledge that the limitations of our study should be considered. First, this study had a single-center and retrospective design. Second, the small sample size may have limited the generalizability of the conclusions. Third, a longer follow-up period is needed to assess the long-term efficacy of surgery.

For CICAO, the timing of recanalization and choice of surgical approach needs to be fully evaluated and carefully decided. For types A and B, both hybrid surgery and EI can achieve recanalization with high success and low complication rates. For patients who cannot tolerate surgery under general anesthesia, EI is a better choice. For type C, the more appropriate choice is hybrid surgery, which can improve the recanalization success rate by removing plaques, pulling out clots, and treating tandem lesions. Simultaneously, CEA has also created artificial stumps that facilitate the endovascular treatment (9). For type D patients, hybrid surgery and endovascular therapy are not recommended because recanalization attempts are often futile. Overall, there was no difference in the rates of complications or restenosis between hybrid surgery and endovascular treatment.

## Data Availability

The original contributions presented in the study are included in the article/Suplementary Material, further inquiries can be directed to the corresponding author/s.
